# Nutritional Description of Foods with Low- and No-Calorie Sweeteners in Spain: The BADALI Project

**DOI:** 10.3390/nu14132686

**Published:** 2022-06-28

**Authors:** Marta Beltrá, Héctor Tomás, Juan C. López, Fernando Borrás, Ana B. Ropero

**Affiliations:** 1 Institute of Bioengineering, Miguel Hernández University, 03202 Elche, Spain; beltra@umh.es (M.B.); hector.tomas01@goumh.umh.es (H.T.); jclopezdelpueblo@gmail.com (J.C.L.); 2 Department of Statistics, Mathematics and Informatics, Miguel Hernández University, 03202 Elche, Spain; f.borras@umh.es

**Keywords:** non-nutritive sweeteners, no calorie sweeteners, polyols, intense sweeteners, added sugar, nutrition claims, health claims, regulation compliance, nutrient composition, food products reformulation, beverages

## Abstract

The use of low- and no-calorie sweeteners (LNCS) in foods has increased in recent years in response to the negative effects of free sugar on health. However, the health impact of LNCS is still unclear. Studies of the prevalence of LNCS in foods have been published previously, including in Spain. However, the use of health (HCs) and nutrition claims (NCs) to promote these foods and a full nutritional characterization are largely lacking. For this purpose, we used the BADALI database with 4218 foods present in the Spanish market. Our results show that 9.3% of foods have LNCS (including both intense and polyols). Sucralose and acesulfame K were the intense sweeteners most frequently used (52.4% and 48.2%, respectively), whereas maltitol was the preferred polyol (20.3%). Of all foods with LNCS, 30% also had added sugar. Many more foods with LNCS presented HCs and NCs than those without. Sugar was the nutrient most frequently claimed in NCs for LNCS-containing foods, whereas vitamins were for those without these sweeteners. NCs compliance with regulation was similar in both conditions (60.1% for foods without and 63.9% for foods with LNCS). As expected, foods with LNCS had less total sugar content and energy. Surprisingly, the nutrient profile of yogurts with LNCS changed completely: less total and saturated fat, whereas more proteins and sodium. Biscuits with LNCS contained more fibre. The results of our study reveal that the prevalence of LNCS is becoming high in some food types in Spain and that foods containing LNCS are more frequently promoted with HCs/NCs. In addition, it confirms the general reduction in energy and sugar content expected in foods with LNCS. Furthermore, it suggests a reformulation of products beyond sugar content.

## 1. Introduction

According to the World Health Organization (WHO), “a high level of free sugars intake is of concern, because of its association with poor dietary quality, obesity and risk of noncommunicable diseases” [1]. In the same document, WHO suggests a reduction of the intake of free sugars below 5% of total energy intake [1]. Recently, the European Food and Safety Authority (EFSA) stated: “the intake of added and free sugars should be as low as possible in the context of a nutritionally adequate diet” [2]. The WHO definition of free sugars “include monosaccharides and disaccharides added to foods and drinks by the manufacturer, cook or consumer, and sugars naturally present in honey, syrups, fruit juices and fruit juice concentrates” [1]. A more extended definition was developed by Public Health England in 2018 including sugars in vegetables and fruit purées and pastes [3].

Substitution of free sugars by low- and no-calorie sweeteners (LNCS) in processed foods has been ongoing for some decades now in order to ensure sweetness. The percentage of the population consuming LNCS varies greatly among studies. A recent work in Spain reported that as many as 79% of adults do so on a daily basis [4]. Similar results were obtained in Portugal by the same authors [5]. In USA, a publication in 2017 showed that 41.4% of adults consumed LNCS, whereas this figure was 25.1% among children [6]. In Brazil, the value was much lower (13%) [7].

The intake of LNCS or the purchase of non-sugar-sweetened foods has increased in recent years [8,9,10,11,12]. The prevalence of LNCS in foods and drinks has also increased, according to studies in several countries [13,14,15]. This increase may have been propelled, at least in some countries, by taxation and other policies aimed at decreasing the intake of sugar-sweetened foods [16,17]. It is expected that the global market for LNCS will keep increasing around 5% annually according to different estimates [18,19].

Foods with more than 10% of added polyols must include the warning “excessive consumption may produce laxative effects”, according to European Union (EU) regulation [20]. In addition, acceptable daily intake (ADI) for most non-nutritive sweeteners (NNS) has been established [21]. Thus far, most studies performed in several countries conclude that the intake of NNS does not exceed the ADI, although with some warnings [7,22,23,24,25,26]. However, as the use of NNS is expected to increase, surpassing the individual ADI may be possible in the near future.

In spite of the efforts of the authorities to ensure that only safe LNCS are authorised, their health effects are still unclear [27]. Some of the outcomes are contradictory depending on the type of study performed, whether randomized controlled trials or cohort/case–control studies [27]. An example is the observed effects on body weight [27].

Evidence against the use of LNCS is accumulating. A recent publication showed that maternal NNS intake during pregnancy was associated with increased childhood body mass index (BMI) z-score and body fat from birth to teenage years [28]. Studies suggest that NNS may contribute to the development of metabolic syndrome and insulin resistance [29,30,31]. A systematic review and meta-analysis published in 2017 found that consumption of NNS was associated with hypertension, type 2 diabetes and cardiovascular events [30]. LNCS also interfere with gut microbiota composition [29,31,32].

In consequence, some public institutions do not recommend the use of LNCS. The USA Scientific Report of the 2015 Dietary Guidelines Advisory Committee advises against replacing added sugars with LNCS due to the minimal evidence regarding long-term effects [33]. The Pan American Health Organization Nutrient Profile Model include foods with non-sugar sweeteners as not healthy. This is based on the rationale that “habitual use of sweet flavours (sugar-based or not) promotes the intake of sweet food and drinks, including those that contain sugars” [34]. The WHO Regional Office for Europe Nutrient Profile Model does not recommend non-sugar sweetened drinks for children [35]. In some countries, the taxation policies for sugar-sweetened drinks also include those with LNCS [36].

The prevalence of LNCS in foods has been studied in recent years in a good number of countries, including Spain (see Discussion). However, to our acknowledge, a full nutritional characterization of LNCS-containing products has not been performed. It is assumed that replacing added/free sugar with LNCS would result in a decrease in sugar content and probably energy. The re-formulation of products beyond sugar replacement is a possibility that has not been explored. In addition, the use of health (HCs) and nutrition claims (NCs) to attract consumers’ attention to LNCS-containing products has been very poorly studied [37,38]. Therefore, the aims of the present work are: (1) to analyse the prevalence of LNCS in a foods sample of the Spanish market; (2) to study the use of HCs and NCs to promote foods with or without LNCS; and (3) to nutritionally characterize these foods.

## 2. Materials and Methods

### 2.1. BADALI Database of Food Products Available in the Spanish Market

The data used in this work come from the BADALI database project [39,40]. Data were collected from 2017 to 2022. Details about the food and brand selection process can be found in Ropero et al., 2020 [41]. In short, the information used in this study was obtained from the manufacturers’ web pages, including the nutrient composition, ingredients, HCs and NCs. This information was extracted by the researchers and inconsistent data were not used for further analysis. Fresh foods were poorly represented in the database, the main exception being fish and seafood (included in G6). For the purpose of this study and in order to reduce heterogeneity, foods were classified following similarities in the main ingredients: G1—Cereals—no free sugar; G2—Cereals—sweet derivatives; G3—Cheese and other dairy products; G4—Dairies and substitutes; G5—Fats and oils; G6—Fish, meat and seafood; G7—Fruits, legumes, nuts, seeds and vegetables; G8—Non-alcoholic drinks; G9—Precooked and ready-to-eat food; G10—Sauces; G11—Snacks; G12—Sweets and chocolates (Supplemental material Table S1). Specific food types of special relevance for the purpose of this work were also studied: biscuits (G2—cereals—sweet derivatives), chocolates (G12—sweets and chocolates), fruit drinks (G8—non-alcoholic drinks), jams (G12—sweets and chocolates), soft drinks (G8—non-alcoholic drinks) and yogurts (G4—dairies and substitutes). When data were provided separately for foods and drinks, the latter included milk and milk drinks (G4), vegetable drinks (G4) and non-alcoholic drinks (G8).

When the ingredient list was not present on the manufacturer’s web page, it was obtained from two online supermarket websites: Alcampo and Hipercor. This was used only for products collected from 2020 to 2022. This was not the case for those collected in 2017–19 because at that time only the manufacturers’ webpages were used. It was not conducted later on because products may have changed in the time lapsed. A previous work was performed validating the information on those two online supermarkets, as follows. A sample of 107 foods from 22 brands were used. The ingredient list found on the website of those supermarkets was compared with the one on the manufacturer’s webpage. We found that only one in ten products presented significant differences (11.4% in Alcampo and 9.5% in Hipercor). In addition, 86% of foods presenting significant differences were the same for both online supermarkets.

### 2.2. Presence of LNCS and Added Sugar

Foods in the database were classified according to the presence or absence of LNCS. LNCS analysed in this work were both intense: acesulfame K, aspartame, cyclamate, saccharine, steviol glycosides, sucralose and neohesperidin DC; and polyols: maltitol, isomalt and sorbitol. No other LNCS were detected. Only LNCS used with the purpose of providing sweet taste were surveyed. The use of polyols as additives with other functions was not examined in this work (sorbitol added as humectant or any other function, nor glycerol).

A food product had added sugar when any mono or disaccharide was added in order to increase the sweetness, including fructose, glucose, dextrose, sugar and malt extract. However, maltodextrin, polydextrose and lactose were not regarded as added sugar due to their low sweetness. Nor were fresh or dried fruit purée or pulp.

### 2.3. Health and Nutrition Claims Analysis

Only claims presented to costumers on the brand webpages as text were analysed, including text in the images of packages. When the latter could not be properly read, images were obtained from other sources.

For HCs, only the prevalence in foods was analysed. Despite the compulsory regulation regarding the use of HCs [42], we found that quite often manufacturers do not comply with it. Therefore, we had to establish some extra criteria to analyse HCs:

- Brand names such as, “vitalinea”, “sveltesse”, “linea”, “vitalday”, “diet”, “devoragras” and “active”, were considered HCs since consumers may perceive a beneficial effect. The same was applied to “digestive”, used to identify specific biscuits.
- Words and phrases such as “healthy”, “take care of yourself in a healthy way”, “helps you take care of yourself” or any mention of pleasure were not regarded as HCs. Nor were sentences such as “suitable for diabetics”, “suitable for weight control diets” or “ideal for balanced diets”.
- Sentences such as “with all the benefits of” any of the ingredients were considered as HCs.
- HCs about individual ingredients of foods were not included in the analysis.
- Mentioning the presence of any kind of microorganisms was not regarded as HCs unless they were described as “probiotics”.

The evaluation of NCs was performed following the methodology described in Ropero et al., 2020 [41]. Some extra criteria had to be established and some modifications were made:

- For the claim “with no added sugar”, it was considered that the food had sugars naturally present if the amount was greater than 0.5 g/100 g or 100 mL, which is the maximum permitted to claim “sugar-free” [42]. We applied the rounding guidelines recommended by the European Commission [43].
- “X % less added sugar” was regarded as a non-authorised NC.
- NCs accompanying HCs were listed as individual NCs. As an example, “it contains zinc, which contributes to normal carbohydrate metabolism” was listed as one HC and one NC for zinc.
- Mentioning the amount of a nutrient somewhere else other than in the nutrition declaration did not constitute a NC.

As previously reported, NCs were classified as authorised when they were listed in the Annex of Regulation 1924/2006. The rest were considered as non-authorised [41,42]. In “others”, the NCs “naturally/natural” and “reduced” were included. Some of the NCs could not be evaluated because of the lack of information. Therefore, they were not considered when compliance was analysed. Non-authorised NCs were all incorrect, since only those authorised are permitted in foods [42].

### 2.4. Nutrient Composition of Matched Products

Foods with LNCS were matched with their non-LNCS counter partners. Since the number of pairs in the database was small, more foods were recruited from other manufacturers’ and online supermarkets’ web pages. These new products were only used for this analysis. The following criteria were followed:

- Both products must be of the same brand.
- Only one-to-one matches were used.
- The most similar LNCS-free product was chosen among all the alternatives consumers were presented with. Therefore, an exact match was not required.
- The LNCS-free product must have added sugar.
- The LNCS-containing foods may or may not have added sugar.
- No LNCS-free alternative was assigned for brands with only LNCS-containing products.

### 2.5. Statistics

The Kruskal–Wallis H test (sometimes also called the “one-way ANOVA on ranks”) is a rank-based nonparametric test that can be used to determine if there are statistically significant differences between two or more food groups of an independent variable on a continuous or ordinal dependent variable. Nonparametric ANOVA has no assumption of normality of random error but the independence of random error is required. The chi-square test of homogeneity was used to determine whether different columns (or rows) of data in a table come from the same population or not (i.e., whether the differences were consistent with being explained by sampling error alone). The statistical analysis of the application data in this work was performed with Microsoft Excel and Google Colab with Jupyter Notebooks, libraries scikit-learn 0.22.2.post1, Pandas v0.25.3, and Matplotlib Python v3.2.0. The significance level was set as *p* < 0.05 in all statistical analysis.

## 3. Results

### 3.1. Presence of LNCS and Added Sugar

A total of 4218 products were collected and classified into 12 food groups (Table 1 and Table S1). The ingredient list was available for 3558 foods. Of all these, 9.3% contained any LNCS (intense sweeteners or polyols), which were heterogeneously distributed among food groups. LNCS were present in 5.7% of foods and in 29.3% of drinks (Table 1). Only 6 out of 12 food groups had any food with LNCS, being significant only in four of them (G2, G4, G8 and G12). As shown in Table 1, as many as 41.6% of non-alcoholic drinks contained LNCS. They were exclusively soft drinks and fruit drinks (except for one), with 78.1% and 51.5% prevalence, respectively (Table S2). Dairies and substitutes (G4) also presented a high proportion of foods with LNCS, mainly yogurts/fermented milk (Table 1 and Table S2). LNCS in sweets were mainly in chocolates and jams (Table S2). As for sweet cereal derivatives, most products with LNCS were biscuits (Table S2).

**Figure 1:**
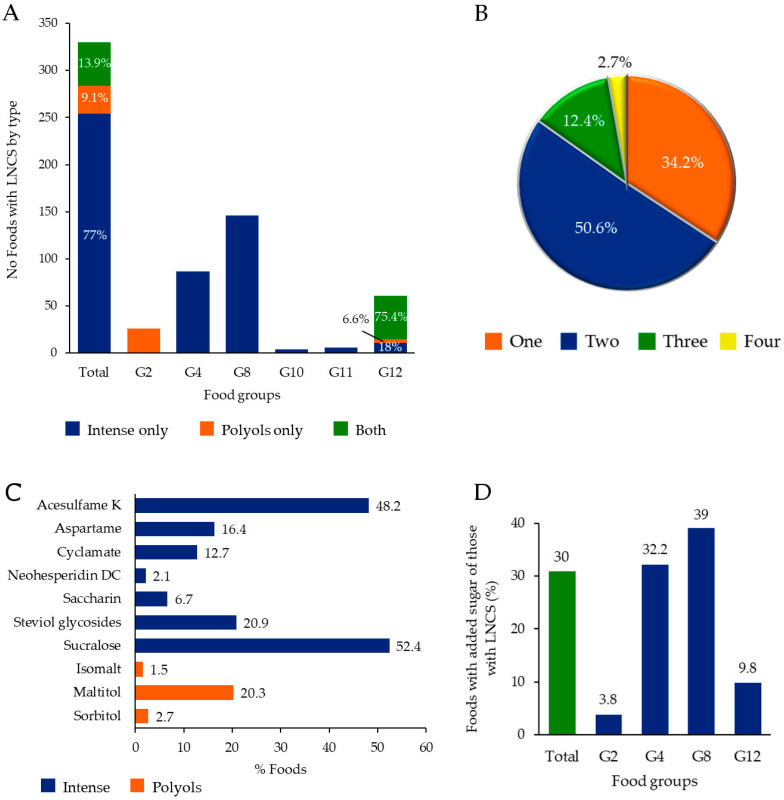
Characterization of foods with LNCS. (**A**) Distribution of different types of LNCS in the total population and by food group. (**B**) Percentage of foods with a different number of LNCS per food. (**C**) Percentage of foods with each individual LNCS in foods. (**D**) Percentage of foods with added sugar among those with LNCS. Note: snacks and sauces are not included in the figure as individual food groups because of the low number of foods with LNCS; nonetheless, they are included in “Total”. Food group identifiers: G2 = cereals—sweet derivatives; G4 = dairies and substitutes; G8 = non-alcoholic drinks; G10 = sauces; G11 = snacks; G12 = sweets and chocolates.

Intense LNCS were the most frequently used sweeteners. They were added alone to 7.1% of foods, which represented 77% of the total with LNCS (Figure 1A). They were present throughout all food groups, except for G2 (cereals—sweet derivatives). Polyols alone were only used in 0.8% of foods (9.1% of foods with LNCS), all of them cereals—sweet derivatives (G2) and sweets—chocolates (G12) (Figure 1A, Table S2). Curiously enough, both types of sweeteners were added in 1.3% of foods (13.9% of foods with LNCS), all of them sweets (G12) (Figure 1A, Table S2).

The use of two LNCS per food was predominant, whereas around one-third of the sample contained only one (Figure 1B). The maximum number of LNCS in a single product was four. The most frequently used intense LNCS were sucralose and acesulfame K, whereas it was maltitol for polyols (Figure 1C).

Since the proportion of products with LNCS were significant only in four food groups, the rest of the analysis was performed only on those food groups. These were: G2—cereals—sweet derivatives; G4—dairies and substitutes; G8—non-alcoholic drinks; and G12—sweets and chocolates.

Sugar was added to 30% of all foods with LNCS (Figure 1D), which represented 2.8% of all foods studied. The addition of sugar was most frequent in non-alcoholic drinks (G8) and dairy and substitutes (G4) (Figure 1D). Regarding specific food types, nearly half of the soft drinks, almost one-third of fruit drinks and yogurts/fermented milk had added sugar (Table S2).

### 3.2. Health and Nutrition Claims

**Figure 2:**
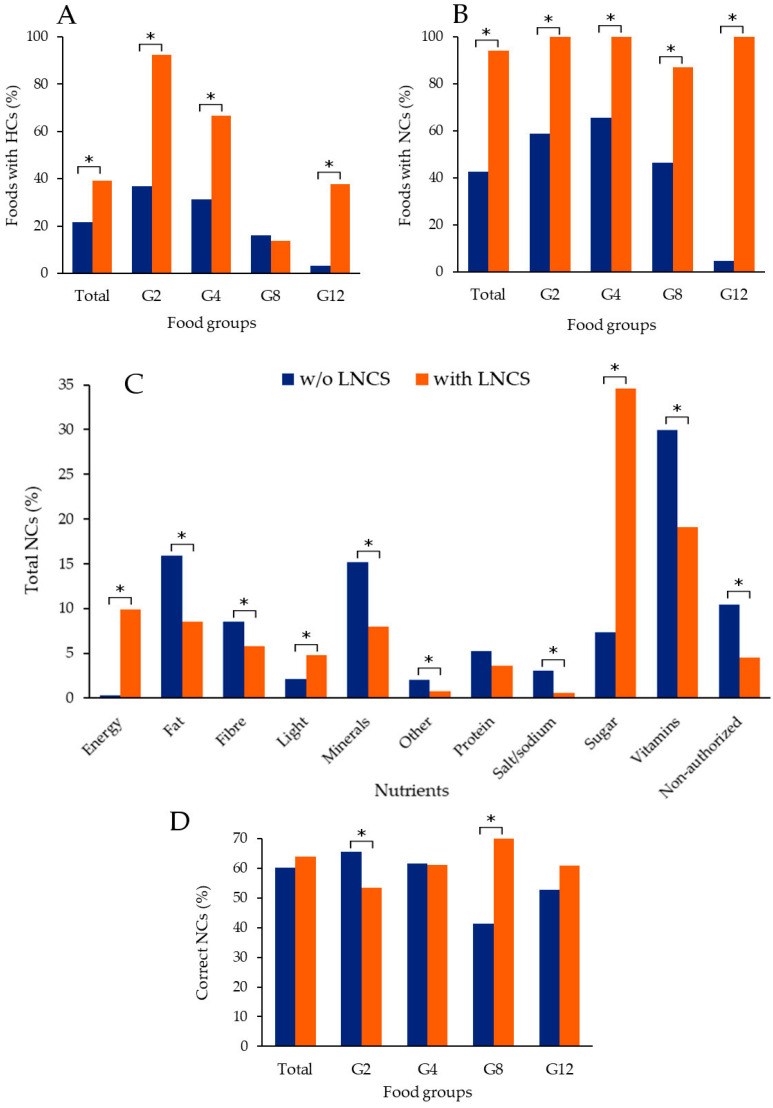
Presence of health (HCs) and nutrition claims (NCs) in foods with and without (w/o) LNCS. Total is the sum of HCs/NCs in G2, G4, G8 and G12. (**A**) Percentage of foods with HCs. (**B**) Percentage of foods with NCs. (**C**) Percentage of total NCs by nutrient. (**D**) Percentage of correct NCs: total and by food group (only evaluated NCs were included). Values represented in this figure can be consulted in Tables S3 and S4. * Statistically significant differences according to *p* < 0.05. Food group identifiers: G2 = cereals—sweet derivatives; G4 = dairies and substitutes; G8 = non-alcoholic drinks; G12 = sweets and chocolates.

When the use of HCs and NCs was analysed, important differences were observed between foods with and without LNCS. The prevalence of HCs was much higher among foods with LNCS, except for non-alcoholic drinks (G8) (Figure 2A). As for NCs, all foods with LNCS in three of the four food groups presented claims. Differences between the two conditions for NCs were statistically significant for all food groups (Figure 2B).

When nutrients claimed were analysed, important differences were also observed (Figure 2C and Table S4). Sugar was the main nutrient claimed in LNCS-containing foods. In fact, the prevalence was more than 4-fold than in foods without LNCS (Figure 2C). Sugar was predominant in food groups G2 (cereals—sweet derivatives) and G12 (sweets and chocolates) (Figure S1). As for foods without LNCS, vitamins were the nutrients most claimed, followed by fat and minerals (Figure 2C). Dissimilarities were also observed for other nutrients, as well as for non-authorised NCs (Figure 2C). The most striking difference was for energy: while only a few foods without LNCS had a claim about energy (0.3%), the value was 9.9% among those with LNCS. Most NCs about energy were in G8 (non-alcoholic drinks) and G12 (sweets and chocolates). All the data on the distribution of NCs by nutrients and food groups can be consulted in supplemental material: Tables S3 and S4 and Figure S1.

Regarding compliance with European Regulation [42], it was similar for foods with or without LNCS (Figure 2D and Table S3). While NCs on foods with LNCS were more compliant with regulation in G8 (non-alcoholic drinks), the opposite was the case for G2 (cereals—sweet derivatives) (Figure 2D and Table S3). When compliance was analysed by nutrients, it was higher in foods with LNCS for fibre, fat, proteins and vitamins (Table S4).

### 3.3. Nutrient Composition

Since substitution of added sugar with LNCS is expected to affect sugar content and probably energy, we decided to confirm this hypothesis in our sample. Possible changes in other nutrients were also examined. In order to minimize the heterogeneity within food groups, only specific food types with at least 20 foods per condition were analysed (Table 2).

Interesting results were observed (Table 2). As expected, all food types with LNCS presented a significant decrease in energy and total sugar content. The extent of the energy decrease was higher when sugar was the main nutrient present, as it is the case for fruit drinks, jams and soft drinks (a 51%, 78% and 94% reduction, respectively). The effect on biscuits was very slight (9% reduction) and a bit higher for yogurts (38.3). Consequent to the changes in sugar content, carbohydrates also decreased in all food types, except for biscuits.

Surprisingly, yogurts with LNCS presented differences in all nutrients studied: 87% less total fat, 93% less saturated fat, 21.2% more proteins and 18.2% more sodium (Table 2). The striking decrease in fat was used to promote these yogurts. In fact, 70.6% of them presented NCs about fat (48 of 68). Similarly, biscuits with LNCS contained more fibre (Table 2) and 95% of them had NCs about fibre (21 of 22). Sodium content was higher in soft drinks, which may be due to the use of cyclamate in some of them (25 of 75), usually added as the sodium salt. Jams with LNCS had more fibre (Table 2).

The presence of added sugar in an important percentage of foods and drinks with LNCS may cause different nutrient profiles. Therefore, we explored this possibility and the results are shown in Table 2. This analysis was only performed for fruit, soft drinks and yogurts because of the small sample for jams and biscuits (Table 2). Sugar was higher for foods with added sugar in all three food types studied (Table 2), whereas heightened energy and carbohydrates was only seen in soft drinks and yogurts (Table 2). The rest of nutrients were mostly unaffected (Table 2).

These results suggest a reformulation of certain food types beyond mere sugar replacement. We decided to confirm this hypothesis in a more homogeneous sample. Therefore, foods with LNCS were matched with their non-LNCS counter partners of the same brand. Since the number was small, pairs of foods were soughtin the manufacturers’ and online supermarkets’ web pages. This rendered equal sample size for both conditions, minimizing the effect of unequal samples on statistics. In addition, the reformulation of products may be more frequent in brands offering both types of products.

We were not able to obtain a significant number of matched products for soft drinks because only a few with LNCS had no added sugar. In fact, many brands added LNCS to all their drinks (including the “original” version with added sugar). We also observed that some yogurt brands did not have products with added sugar or LNCS, but instead used fruit to sweeten the products (as pieces or purée).

The results obtained with matched products confirmed those observed with the unmatched sample (Table 2—highlighted rows). Therefore, the reformulation of biscuits and yogurts was also evident under these controlled conditions. Since we obtained a significant number of matched chocolates, they were also analysed. As expected, chocolates with LNCS presented a striking reduction in sugar content and a slight decrease in energy values (10.6%). The rest of nutrients were not affected (Table 2).

## 4. Discussion

The results presented in this work show that foods and drinks with LNCS are quite frequent in the Spanish market. The use of HCs and NCs is higher in LNCS-containing foods. Nutrients claimed in NCs are different depending on the presence or absence of LNCS, whereas compliance is very similar. When the nutritional composition of foods was analysed, sugar content and energy values were lower for foods with LNCS, as expected. However, we observed some unexpected changes in yogurts and biscuits.

### 4.1. LNCS in Foods

Studies on the prevalence of LNCS in foods and drinks in several countries, including Spain, have been published in recent years [4,5,13,14,15,17,38,44,45,46,47,48,49,50,51,52,53,54,55,56,57,58,59]. Despite differences, some general conclusions may be drawn, most of them shared by our own results. Since the presence of LNCS in foods is increasing with time [13,14,15], we review here only those works with data recorded in the last decade (≥2012).

Total prevalence of LNCS in foods and drinks vary from around 1% in Australia to up to 16% in Colombia, with the exception of Chile with 38–55.5%, depending on the study [5,13,14,17,38,44,47,48,49,50,51,52,53,54,55,58,59]. We obtained 9.3% in a sample of 3558 foods, well within the international range and very similar to the 10% obtained in 1164 foods from the ANIBES study [44]. The presence of LNCS in total or non-alcoholic drinks (dairies are usually not included here) vary between 10% in Hong-Kong to 41.1% in Colombia, with 72–83% in Chile and less than 1% in Australia [5,13,14,15,17,38,47,49,50,53,55,56,58]. Our results are similar to those obtained in Brazil [38]. Non-alcoholic drinks is generally the food group with the highest proportion of LNCS in all countries studied, including our work, and 2–5.5 fold higher than in foods [4,5,13,17,38,44,45,49,53,55,57,58,59]. Our results show that as many as 78.1% of soft drinks had LNCS. In fact, according to our experience in this publication, some brands do not offer LNCS-free drinks, whereas others offer only a few. Other food groups, such as dairies, sweets and desserts, chocolates, processed fruit (jams) and cereal products have also a significant proportion of LNCS [5,13,14,17,38,44,45,46,50,52,53,55,57].

Among intense sweeteners, sucralose and acesulfame K are generally the most frequently used, also in Spain and in our work [5,13,14,15,17,38,44,45,46,52,53,55,56]. In fact, they are the most consumed LNCS among the Spanish population [4]. However, in some works steviol is becoming a major LNCS [13,14,51,55]. Aspartame is also one of the most used in some countries such as Italy, Argentina and Peru [23,24]. Among polyols, sorbitol is the most frequent, whereas the use of maltitol is also significant [4,5,13,38,46,48,52,53,56]. In our database, maltitol was the main polyol used, as well as in the 2019 publication from Spain [45]. The prevalence of sorbitol in the present study is very low. The combination of intense and polyols is becoming quite frequent, even more so than polyols alone [13,38,53]. Our data show that one or two LNCS per food were predominant, with a small proportion having more than two. Similar results were obtained in previous works [17,45,46,53].

The presence of added sugar in LNCS-containing foods was significant in our work, although lower than previously reported in Spain (30% vs 51% respectively as calculated from data in reference [44]). Non-alcoholic drinks and milk/dairy products were two food groups with the highest prevalence in both studies [44]. Publications in other countries have shown a similar tendency, whereas in Brazil, as many as 83% of foods and drinks with LNCS also contained added sugar [13,15,38,48,49,51,53,56]. This high proportion may be the consequence of adding LNCS to the “original” versions, particularly of drinks. In fact, it seems that to the two initial options, either with added sugar or with LNCS, a quite potent third one has been incorporated with both. One reason for this shift may be reducing taxes in those countries taxing sugar sweetened-drinks. Another may be the consumers’ awareness of the negative impact of added sugar to health. As a consequence, consumers accepting added sugar are involuntarily consuming also LNCS. This unawareness contributes to the increase in LNCS intake among the population. In fact, studies of LNCS based on questionnaires completed by the study participants may be missing part of the actual intake of LNCS due to the operation of adding LNCS to original drinks.

Some of the differences observed in the studies reviewed may be due to the different classification of non-sugar sweeteners. In fact, depending on the publication, polyols are not listed, included among no-calorie or as calorie sweeteners. In addition, the use of polyols as additives with functions other than sweeteners are included in some works (this is not the case in the present work). Different definitions of the food groups is another reason for the diversity in results, along with the fact that some food types were not present in our database, such as chewing gum, food supplements and substitutes, energy drinks, low alcohol content drinks, tabletop sweeteners or ice creams.

### 4.2. Health and Nutrition Claims

To our knowledge, only two studies were previously published on nutrition/health claims in products with LNCS: one in Brazil and one in Canada [37,38]. The prevalence in Brazil, with 310 foods with LNCS, was lower than in our case [38]. The authors observed around 50% of non-alcoholic drinks with LNCS carrying HCs/NCs, including dairy drinks (yogurts included, but no vegetable drinks) [38]. In our case, as many as 87% and 100% of dairies and non-alcoholic drinks, respectively, had NCs. Our values for HCs dropped to 14% and 67%, respectively, for these two food groups. As for solid foods, all of them presented NCs, while 38–92% had HCs.

Sugar-specific claims had the highest prevalence in LNCS products in the present work. A study in Canada showed a similar tendency, although the calculations were made differently [37]. The authors observed that LNCS use was more prevalent among products with sugar claims (30%) compared with products without these claims (5%) [37]. Therefore, it seems that sugar claims are undoubtedly linked to LNCS-containing products.

Consumers’ perception and behaviour in response to HCs and NCs have been extensively studied in the last 15 years. Systematic reviews indicate that products bearing an NC have more probability to be chosen and that they can alter adults’ perceptions concerning the content of the products [60,61]. As an example, in a recent paper, a nutrient content claim on a soup increased perceived nutritional quality [62]. Some authors talk about the ‘health halo’ effect, making foods seem healthier than they are [63]. Similar conclusions were drawn from a systematic review published in 2019 on NCs related to sugar and energy content [64]. In fact, in a recent publication, Portuguese participants rated products with sugar claims as healthier and less caloric [65]. As a consequence, these NCs may influence food purchase intentions [64].

The positive effect of NCs and HCs is particularly important for LNCS-containing products because consumer’s perception of these sweeteners is not generally positive [66,67]. Consumers’ concern about LNCS are particularly about artificial sweeteners. The Label Insight Shopper Trends Survey in 2017 in the USA observed that 44% of consumers reported that they avoid them when shopping for food products [68]. In another study by Mintel, more than 4 in 10 consumers who reported avoiding artificial sweeteners, did so mainly because of concerns about health [68]. In the 2018 Global Sweetener Report, only 18% of consumers in the U.S. rated artificial sweeteners as safe [68]. Therefore, NCs and HCs, particularly those related to sugar and energy, may attract consumers’ attention to these products and away from the presence of LNCS.

The compliance with regulation observed in this work was higher than previously reported by our own group in 2020 [41]. However, in the present study only four food groups were studied. Compliance was not affected by the presence or absence of LNCS, but depended on the food group. This is the first analyses of NC compliance in foods with LNCS.

### 4.3. Nutrient Composition

To our knowledge, this is the first full nutritional characterization of products with LNCS comparing them with those without these sweeteners. A previous work published in 2018 analysed sugar density of products in four countries (Australia, Mexico, New Zealand and USA) [50]. As expected, they showed that the presence of NNS was associated with lower mean total sugar density in both drinks and foods. Similar results were obtained in non-alcoholic drinks in Slovenia [15]. These authors also studied energy values and found reductions, particularly in soft drinks [15]. All our results are in agreement with these two publications. In addition, we also obtained energy reduction in biscuits, jams and yogurts. The reduction in sugar and energy in products with LNCS is used by manufacturers to promote their products. In fact, claims about sugar and energy presented the highest difference between foods with and without LNCS.

Our observation that replacement of added sugar with LNCS produced a small energy reduction in biscuits and chocolates (less than 10%) is interesting. Both foods are high-energy foods and sugar is not the main energy contributor. In the case of biscuits, sugar was replaced by polyols, which are caloric and have a lower sweetness. Since there was no change in any of the other calorific nutrients, the reduction in energy was not nutritionally relevant. Most chocolates with LNCS had both intense and polyols, which may decrease energy values more than only with polyols. However, the main nutrient providing energy in chocolates is total fat and that does not change when sugar is replaced by LNCS. As a result, the decrease in energy is small. Jams also contained polyols, although all of them also had intense sweeteners replacing part of the sugar. As sugar is the main energy contributor in jams, the reduction in energy was very high.

We observed that products with LNCS and added sugar had higher energy and total sugar content than those with only LNCS. The same results were obtained by Dunford and Hafner in five countries [15,50]. Therefore, it appears that there are three situations: (1) with added sugar only, which has the highest total sugar and energy content; (2) with LNCS only, which has the lowest total sugar and energy content; (3) with both, which is in between the other two conditions. As we mentioned before, the use of LNCS and added sugar may result in an involuntarily intake of LNCS, since costumers may assume that sugar-sweetened foods do not contain LNCS.

It was interesting to note an entirely different nutrient profile for yogurts with LNCS, strongly suggesting a reformulation beyond sugar substitution. In fact, the decrease in total and saturated fat may be the result of replacing whole or semi-skimmed with skimmed milk. As mentioned before, the use of NCs in products with LNCS may attract consumers’ attention to these products. Fat seems to be an important target for NCs on yogurts with LNCS, as it decreases so dramatically, both total and saturated.

The increment in fibre content in biscuits with LNCS may be due to an increased use of whole cereals or added fibre. This is also used by manufacturers to promote their biscuits with NCs. The use of whole cereals is an improvement since they also provide higher amounts of vitamins and minerals. However, the addition of fibre to refined cereals has only a partial effect on the nutritional improvement of biscuits.

Therefore, it appears that manufacturers not only replace sugar with LNCS, but they also reformulate some products with other purposes. In spite of the improvements some of these reformulations may cause, we must remember that sweet biscuits will remain unhealthy because of their high fat and energy content. In all cases, the substitution of added sugar by LNCS brings about health effects that are still largely unclear.

### 4.4. Strengths and Limitations

The present work has some important strengths:

- This is the first paper published studying the use of HCs/NCs on LNCS-containing foods sold in the Spanish market;
- Foods from all groups were analysed, which provided an overview of the Spanish market;
- More than 4200 foods were analysed and the number of foods per group was significant;
- Data were collected following criteria completely unrelated to the aim of this study or the targeted population and, as a consequence, our results lack any bias on food choice. The only exception is the analysis of matched products in order to have a significant sample;
- Data were collected several years after Regulation (EC) No 1924/2006 on nutrition and health claims was fully in force [43];

Our work has a few important limitations:

- Selection of brands did not follow criteria based on customers’ purchases or the most popular products;
- Data collected were reliant on the accuracy of the information provided on the manufacturers’ webpage;
- The 4218 foods analysed may not be representative of the Spanish market due to the huge number of foods available;
- Only LNCS used for the purpose of sweetening foods were analysed;
- Some of the products displayed 0 g salt/sodium, which could be wrongly rounded. The EC published a guidance document with rounding instructions, but it is not compulsory [44].
- The number of products for the matched comparative study was low due to the restrictive conditions applied.

## 5. Conclusions

The results of our study reveal that LNCS are quite frequent in the Spanish market. In fact, some brands elaborate all their products with LNCS and alternatives with only added sugar are not available. In addition, many of the “original” versions with added sugar already have LNCS. This is of particular concern because this may induce the involuntary intake of LNCS when consuming foods with added sugar. The use of claims to draw consumers’ attention to these products and away from the actual LNCS, may contribute to mask the presence of these sweeteners. Therefore, consumers may overlook their presence and involuntarily increase intake. The reformulation of products with LNCS to improve them is a welcoming side effect. However, consumers may perceive them healthier than they actually are. Above all, we must consider that the role of LNCS on health is still unclear, with some evidence of negative effects. Therefore, we believe that the use of LNCS should be limited.

## Figures and Tables

**Table 1:** Foods included in the study and prevalence of low- and no-calorie sweeteners (LNCS): total and by food group.

Food Groups	No Foods		Foods with LNCS	
	Total	Ingredient Information	No	% Within the Food Group
Total	4218	3558	330	9.3
Foods	3646	3016	171	5.7
Drinks	572	542	159	29.3
G1—Cereals—no free sugar	375	280	0	0
G2—Cereals—sweet derivatives	374	310	26	8.4
G3—Cheese and other dairy products	244	219	0	0
G4—Dairies and substitutes	570	509	87	17.1
G5—Fats and oils	36	30	0	0
G6—Fish, meat and seafood	623	357	0	0
G7—Fruits, legumes, nuts, seeds and vegetables	449	406	0	0
G8—Non-alcoholic drinks	376	351	146	41.6
G9—Precooked and ready-to-eat food	246	242	0	0
G10—Sauces	174	165	4	2.4
G11—Snacks	285	232	6	2.6
G12—Sweets and chocolates	466	457	61	13.3

**Table 2:** Energy and nutrient density of specific food types according to the presence/absence of LNCS and/or added sugar. Values in 100 g or 100 mL.

Food Types	LNCS	Added Sugar	Energy (kcal)			Protein (g)			Carbohydrates (g)			Sugar (g)		
			*n*	Median (IR)	*p*-Value	*n*	Median (IR)	*p*-Value	*n*	Median (IR)	*p*-Value	*n*	Median (IR)	*p*-Value
Biscuits	w/o	--	121	463 (451; 483)	<0.001 *	121	6.3 (5.7; 7.8)	0.825	121	66 (63; 70)	0.267	120	22 (19; 28)	<0.001 *
	with	--	22	426 (412; 439)		22	6.8 (5.9; 7.2)		22	65 (63; 68)		22	0.6 (0; 1.6)	
Biscuits-matched	w/o	--	40	474 (451; 497)	<0.001 *	40	6 (5.2; 6.6)	0.122	40	67 (63; 72)	0.066	39	25 (20; 30)	<0.001 *
	with	--		432 (415; 454)			6.8 (5.6; 7.3)			65 (63; 68)			1.2 (0.6; 2.8)	
Chocolates-matched	w/o	--	32	548 (541; 567)	<0.001 *	32	7.7 (5.4; 11)	0.394	32	44.5 (32.5; 55.3)	0.819	30	43.5 (27.5; 54)	<0.001 *
	with	--		490 (476; 513)			8.2 (6.2; 11)			45 (33.8; 52)			1.2 (0.5; 7.1)	
Fruit drinks	w/o	--	66	47 (45; 50)	<0.001 *	66	0.2 (0; 0.3)	0.059	66	11.3 (10; 12)	<0.001 *	66	11.1 (9.8; 11.8)	<0.001 *
	with	--	70	23 (19; 26)		70	0.1 (0; 0.3)		70	5.3 (4.4; 6)		70	4.8 (4.2; 5.7)	
	with	w/o	49	23 (18; 26)	0.2	49	0.2 (0; 0.3)	0.02 *	49	5.3 (4.1; 5.8)	0.185	49	4.8 (3.8; 5.2)	0.015 *
	with	with	21	23 (20; 31)		21	0 (0; 0.1)		21	5.4 (4.8; 7)		21	5.3 (4.8; 7)	
Fruit drinks-matched	w/o	--	33	47 (44; 52)	<0.001 *	33	0.2 (0.2; 0.5)	0.445	33	11.3 (10.9; 11.9)	<0.001 *	33	10.9 (9.8; 11.5)	<0.001 *
	with	--		25 (22; 27)			0.3 (0.2; 0.5)			5.4 (5; 6.2)			5 (4.3; 5.4)	
Jams	w/o	--	118	210 (180; 231)	<0.001 *	118	0.5 (0.4; 0.6)	0.846	118	52 (44; 57)	<0.001 *	86	48.5 (42.8; 57.4)	<0.001 *
	with	--	26	47 (47; 74.8)		26	0.5 (0.4; 0.5)		26	17 (14; 17)		26	5.4 (5.2; 5.6)	
Jams- matched	w/o	--	40	199 (185; 226)	<0.001 *	40	0.5 (0.3; 0.5)	0.927	40	48 (44; 55.3)	<0.001 *	40	45 (42.7; 50.5)	<0.001 *
	with	--		47 (40; 51)			0.5 (0.4; 0.5)			14 (11.9; 17)			5.4 (3.9; 5.6)	
Soft drinks	w/o	--	21	36 (32; 41)	<0.001 *	21	0 (0; 0)	0.78	21	8.7 (7.8; 9.8)	<0.001 *	21	8.6 (7.8; 9.8)	<0.001 *
	with	--	75	2 (1; 19)		75	0 (0; 0)		75	0.2 (0; 4.4)		75	0.2 (0; 4.1)	
	with	w/o	39	1 (0.4; 1)	<0.001 *	39	0 (0; 0)	0.567	39	0 (0; 0)	<0.001 *	39	0 (0; 0)	<0.001 *
	with	with	36	19 (15; 20.5)		36	0 (0; 0)		36	4.5 (3.5; 4.9)		36	4.3 (3.4; 4.6)	
Yogurts	w/o	--	143	77 (68.5; 94.5)	<0.001 *	143	3.4 (3; 3.7)	<0.001 *	143	11 (7.8; 13.2)	<0.001 *	143	11 (7.2; 12.5)	<0.001 *
	with	--	68	47.5 (40; 56)		67	4.2 (3.2; 4.8)		68	5.6 (4.6; 7)		68	5.1 (4.4; 6.3)	
	with	w/o	48	43 (40; 54.5)	0.036 *	48	ND	ND	48	5.2 (4.4; 6.1)	<0.001 *	48	4.8 (4.2; 5.7)	<0.001 *
	with	with	20	54 (49.8; 56.5)		19	ND		20	7 (6.4; 7.8)		20	6.5 (5.4; 7.5)	
Yogurts-matched	w/o	--	45	78 (68; 87)	<0.001 *	44	3.3 (2.9; 3.7)	<0.001 *	44	11.7 (11; 13)	<0.001 *	45	11.4 (11; 12.4)	<0.001 *
	with	--		39 (37; 46)			4 (3; 4.3)			5.4 (4.6; 5.9)			5 (4; 5.6)	
**Food Types**	**LNCS**	**Added Sugar**	**Total Fat (g)**			**Saturated Fat (g)**			**Fibre (g)**			**Sodium (mg)**		
			***n***	**Median (IR)**	***p*-Value**	***n***	**Median (IR)**	***p*-Value**	***n***	**Median (IR)**	***p*-Value**	***n***	**Median (IR)**	***p*-Value**
Biscuits	w/o	--	121	18 (15.7; 21)	0.374	121	4.2 (1.8; 9)	0.1	120	3.7 (2.7; 5.6)	<0.001 *	121	280 (200; 380)	0.159
	with	--	22	17 (14.3; 20.8)		22	2.2 (1.5; 6)		22	5.8 (4.5; 9.2)		22	250 (214; 300)	
Biscuits-matched	w/o	--	40	19.5 (15; 23.3)	0.866	39	5 (1.8; 12.9)	0.392	37	3 (2.4; 4)	<0.001 *	39	272 (180; 350)	0.187
	with	--		17 (14.8; 24)			3.6 (1.5; 10.5)			5.3 (4; 9)			228 (116; 300)	
Chocolates-matched	w/o	--	32	36 (33.8; 40.5)	0.788	32	20.5 (18; 22.3)	0.34	0	ND	ND	32	50 (24; 77)	0.448
	with	--		36.5 (34; 40.5)			21 (20; 23.3)			ND			44 (24; 68)	
Fruit drinks	w/o	--	65	0 (0; 0)	0.189	66	0 (0; 0)	0.446	14	ND	ND	66	4 (0; 4)	0.705
	with	--	70	0 (0; 0)		70	0 (0; 0)		12	ND		70	4 (0; 12)	
	with	w/o	49	0 (0; 0)	0.346	49	0 (0; 0)	1	8	ND	ND	49	4 (0; 8)	0.27
	with	with	21	0 (0; 0)		21	0 (0; 0)		4	ND		21	8 (0; 12)	
Fruit drinks-matched	w/o	--	33	0 (0; 0.1)	1	33	0 (0; 0)	1	6	ND	ND	33	4 (0; 4)	0.812
	with	--		0 (0; 0.1)			0 (0; 0)			ND			4 (0; 4)	
Jams	w/o	--	118	0 (0; 0.1)	0.578	86	0 (0; 0)	0.717	81	1.2 (0.8; 1.2)	<0.001 *	85	0 (0; 0)	1
	with	--	26	0 (0; 0.2)		26	0 (0; 0)		23	5.9 (1.3; 5.9)		26	0 (0; 0)	
Jams- matched	w/o	--	40	0 (0; 0.1)	0.81	40	0 (0; 0)	0.711	11	ND	ND	40	0 (0; 0)	0.234
	with	--		0 (0; 0.2)			0 (0; 0)			ND			0 (0; 13)	
Soft drinks	w/o	--	19	ND	ND	21	0 (0; 0)	1	0	ND	ND	21	0 (0; 0)	<0.001 *
	with	--	73	ND		75	0 (0; 0)		7	ND		75	20 (4; 50)	
	with	w/o	39	0 (0; 0)	0.666	39	0 (0; 0)	1	6	ND	ND	39	12 (4; 20)	0.013 *
	with	with	34	0 (0; 0)		36	0 (0; 0)		1	ND		36	36 (11; 52)	
Yogurts	w/o	--	143	2.3 (1.8; 3.7)	<0.001 *	143	1.5 (1.2; 2.4)	<0.001 *	10	ND	ND	143	44 (40; 48)	<0.001 *
	with	--	68	0.3 (0.1; 0.5)		68	0.1 (0.07; 0.2)		12	ND		68	52 (40; 60)	
	with	w/o	48	0.1 (0.1; 0.5)	0.567	48	0.1 (0.07; 0.2)	0.737	9	ND	ND	48	56 (46; 60)	0.156
	with	with	20	0.4 (0.1; 0.7)		20	0.1 (0.08; 0.2)		3	ND		20	46 (40; 60)	
Yogurt-matched	w/o	--	45	1.9 (1.1; 2.2)	<0.001 *	45	1.2 (0.8; 1.4)	<0.001 *	5	ND	ND	45	44 (40; 52)	<0.001 *
	with	--		0.4 (0.1; 0.5)			0.1 (0.06; 0.1)			ND			60 (48; 60)	

--: conditions not considered. ND: not determined because of <20 foods/condition. w/o: without LNCS. * Statistically significant differences according to *p* < 0.05.

## Data Availability

Not applicable.

## Supplementary Materials

The following supporting information can be downloaded at: https://www.mdpi.com/article/10.3390/nu14132686/s1, Table S1: Description of the items included in the food groups; Table S2: Foods included in the study, prevalence of LNCS and added sugar by food type; Table S3: Presence of HCs and NCs in foods with and without (w/o) LNCS; Table S4: NCs presence and compliance, by nutrient. Figure S1: Distribution of NCs by nutrient and food group (total NCs were considered).
