# Supplementary material for: Nutritional Description of Foods with Low- and No-Calorie Sweeteners in Spain: The BADALI Project
Source: Nutrients. 2022 Jun 28;14(13):2686. doi: 10.3390/nu14132686 (PMC9268128; doi:10.3390/nu14132686)
Supplement: Supplementary file 1 [file nutrients-14-02686-s001.zip › nutrients-1780960-supplementary.pdf]

Table S1. Description of the items included in the food groups.

	Food Groups	Foods
**G1**	Cereals - no free sugar	Cereal cakes, cereal flakes, grain cereals, seeds, flour, bran, gluten, germ, bread, breadcrumbs, toasts, croutons, bread slices and sticks, cakes (oatmeal, rice, corn), cereal tortillas, pizza dough, precooked bread, sliced bread, rice, pasta made of cereals, cereals with vegetables, pasta made of legumes; semolina, bulgur, couscous, quinoa
**G2**	Cereals - sweet derivatives (with free sugar)	Sweet biscuits, breakfast cereals, cereal bars, pastries and pastry mix
**G3**	Cheese and other dairy products	All kinds of cheese, including fresh, semi-cured, cured, cottage, whipped, spreads, cream, concentrated milk (evaporated, condensed and powdered)
**G4**	Dairies and substitutes	All kinds of milk, flavoured milk shakes, coffees with milk, fermented milk, dairy desserts (curd, panna cotta, mousse, custard, rice pudding), vegetables drinks (oats, soy, wheat, spelt, rice, coconut, almond, nuts), vegetable alternative fermented and desserts
**G5**	Fats and oils	Butter, margarine, lard
**G6**	Fish, meat and seafood	Sausages, cold meat, luncheon meat, other processed meat, meat derivatives (pate and spreads), fresh and canned fish and seafood, prepared for fish or seafood paella, liver, roe and fish derivatives (surimi and spreads)
**G7**	Fruits, legumes, nuts, seed and vegetables	Dried fruit, dried or canned legumes, natural nuts, seeds, olives, processed vegetables, canned vegetable, mushrooms, processed foods from plant origin with added ingredients (spreads, hummus, tofu crushed tomato)
**G8**	Non-alcoholic drinks	Fruit and vegetable smoothies, juices, nectars, tigernut drinks, coffee, tea, soft drinks, tonic water, soda water, non-alcoholic beer, non-alcoholic sangria. Fruit drinks were considered when a minimum of 6% fruit or juice was present.
**G9**	Precooked and ready-to-eat food *	Ready-to-eat salads, meatloaf, cooked rice, chicken wings, onion rings, battered meat, vegetables, fish and squid, filled and ready-to-eat pasta dishes, churros, couscous, croquettes, falafel, nuggets, fingers, cooked vegetables, spring rolls, paellas, pizzas, vegetable alternative to processed meat (sausages, burgers, cold meat, meatballs), creams, gazpacho
**G10**	Sauces	Bechamel, mayonnaise, fried tomato, vegetable stir fries, ketchup, barbecue, hot, mustard, allioli, bittersweet, soy, pepper, andalouse, burger, caesar, chives, chimichurri, cocktail, curry, boletus, carbonara, pedro ximénez, cheese, yogurt, kebab, pesto, vinaigrette, spicy
**G11**	Snacks	Popcorn, chips (potato, legumes), corn snacks, wheat snacks (sticks), nachos, pork fried crusts, fried vegetables; snacks elaborated with potatos, vegetables or legumes; salty nuts, crackers, salted sunflower seeds
**G12**	Sweets and chocolates	Fruits and vegetable jams, honey, chocolates, chocolate bars, cocoa and derivatives, mix for chocolate products, chocolate snacks, turrón, caramelised nuts and vegetables, fruits in syrup, quince, sweets, fruit spreads

* Processed foods ready to eat with minimum cooking not included in other groups (according to the instructions in the pack) are also included here. Foods in this group have added salt, fat and/or additives.

Table S2. Foods included in the study, prevalence of LNCS and added sugar by food type

Food Types/ No Foods	Total	Ingredient Information	LNCS (%) *	Added sugar (%) *	Intense sweeteners only (%) *	Polyols only (%) *	Both (%) *
**Biscuits**	194	143	22 (15.4)	1 (4.5)	0 (0)	22 (100)	0 (0)
**Chocolates**	178	171	18 (10.5)	0 (0)	0 (0)	4 (22.2)	14 (77.8)
**Fruit drinks**	144	136	70 (51.5)	21 (30)	70 (100)	0 (0)	0 (0)
**Jams**	144	144	26 (18.1)	3 (11.5)	4 (15.4)	0 (0)	22 (84.6)
**Soft drinks**	112	96	75 (78.1)	36 (48)	75 (100)	0 (0)	0 (0)
**Yogurts/fermented milk**	247	211	68 (32.2)	20 (29.4)	68 (100)	0 (0)	0 (0)

* % within the specific food type

Table S3. Presence of HCs and NCs in foods with and without (w/o) LNCS

Food Types	LNCS	No Foods				No NCs				No Incorrect NCs	
		With HCs		With NCs		Total	Evaluated	Correct		Authorized (%)**	Non-authorized (%)**
		No (%)*	*p*-value	No (%)*	*p*-value			No (%)*/**	*p*-value		
**Total**	w/o	283 (21.7)	< 0.001 $^{\#}$	558 (42.7)	< 0.001 $^{\#}$	2105	2092	1258 (60.1)	0.067	615 (29.4)	219 (10.5)
	with	125 (39.1)		301 (94.1)		801	794	507 (63.9)		251 (31.6)	36 (4.5)
**G2**	w/o	105 (37)	< 0.001 $^{\#}$	167 (58.8)	0.002 $^{\#}$	577	576	378 (65.6)	0.045 $^{\#}$	172 (29.9)	26 (4.5)
	with	24 (92.3)		26 (100)		71	71	38 (53.5)		28 (39.4)	5 (7)
**G4**	w/o	132 (31.3)	< 0.001 $^{\#}$	277 (65.6)	< 0.001 $^{\#}$	1248	1238	761 (61.5)	0.91	326 (26.3)	151 (12.2)
	with	58 (66.7)		87 (100)		292	288	176 (61.1)		85 (29.5)	27 (9.4)
**G8**	w/o	33 (16.1)	0.984	95 (46.3)	< 0.001 $^{\#}$	241	240	99 (41.3)	< 0.001 $^{\#}$	103 (42.9)	38 (15.8)
	with	20 (13.7)		127 (87)		312	312	218 (69.9)		90 (28.8)	4 (1.3)
**G12**	w/o	13 (3.3)	< 0.001 $^{\#}$	19 (4.8)	< 0.001 $^{\#}$	39	38	20 (52.6)	0.361	14 (36.8)	4 (10.5)
	with	23 (37.7)		61 (100)		126	123	75 (61)		48 (39)	0 (0)

LNCS: low- and no-calorie sweeteners. HCs: health claims. NCs: nutrition claims. * Values are represented in Figure 2.
** Only evaluated NCs were considered. $^{\#}$ Statistically significant differences according to $p < 0.05$.

Table S4. NCs presence and compliance in foods, by nutrient

Food Types	LNCS	No NCs				
		Total		Evaluated	Correct	
		No (%) *	*p*-value		No (%) **	*p*-value
**Energy**	w/o	6 (0.3)	< 0.001 #	6	5 (83.3)	ND
	with	79 (9.9)		79	51 (64.6)	
**Fat**	w/o	335 (15.9)	< 0.001 #	333	130 (39)	< 0.001 #
	with	68 (8.5)		67	60 (89.6)	
**Fibre**	w/o	180 (8.6)	0.012 #	177	147 (83.1)	0.003 #
	with	46 (5.7)		46	46 (100)	
**Light**	w/o	45 (2.1)	< 0.001 #	45	22 (48.9)	0.390
	with	38 (4.7)		38	15 (39.5)	
**Minerals**	w/o	319 (15.2)	< 0.001 #	319	241 (75.5)	0.721
	with	64 (8)		64	47 (73.4)	
**Other**	w/o	42 (2)	0.019 #	42	18 (42.9)	ND
	with	6 (0.7)		5	0 (0)	
**Proteins**	w/o	110 (5.2)	0.07	108	76 (70.4)	0.004 #
	with	29 (3.6)		28	27 (96.4)	
**Salt**	w/o	65 (3.1)	< 0.001 #	64	59 (92.2)	ND
	with	5 (0.6)		5	5 (100)	
**Sugar**	w/o	154 (7.3)	< 0.001 #	150	54 (36)	0.096
	with	277 (34.6)		273	121 (44.3)	
**Vitamins**	w/o	630 (29.9)	< 0.001 #	629	506 (80.5)	0.025 #
	with	153 (19.1)		153	135 (88.2)	
**Non-authorized**	w/o	219 (10.4)	< 0.001 #	219	0 (0) ##	--
	with	36 (4.5)		36	0 (0) ##	

ND: not determined because of < 25 NCs/condition.* Percentage of the total NCs (2105 for foods w/o LNCS; 801 for foods with LNCS); values are represented in Figure 2C. ** Only evaluated NCs were considered for the analysis. LNCS: Low- and No-Calorie Sweeteners. w/o: without LNCS.
Statistically significant differences according to $p < 0.05$. ## All non-authorized NCs were considered incorrect following Regulation 1924/2006 [42] (see Materials and Methods)

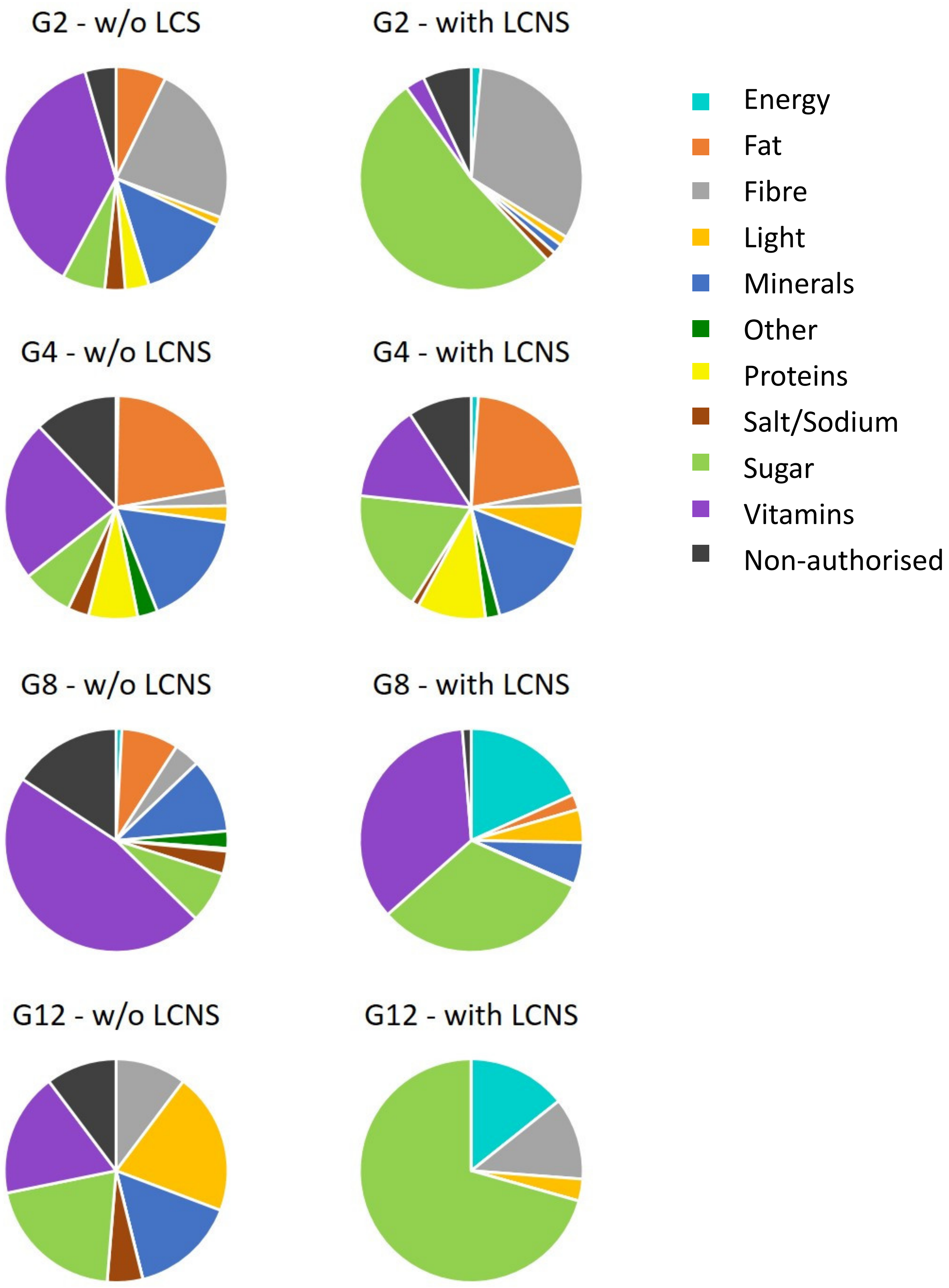

Figure S1. Distribution of NCs by nutrient and food group (total NCs were considered).
